# Postoperative Analgesia after Open Liver Surgery: Systematic Review of Clinical Evidence

**DOI:** 10.3390/jcm10163662

**Published:** 2021-08-18

**Authors:** Paula Dudek, Mateusz Zawadka, Paweł Andruszkiewicz, Remigiusz Gelo, Francesco Pugliese, Federico Bilotta

**Affiliations:** 1 2nd Department of Anesthesiology and Intensive Care, Medical University of Warsaw, 02-097 Warsaw, Poland; pdudek@wum.edu.pl (P.D.); paul.andruszkiewicz@gmail.com (P.A.); remekgelo@poczta.onet.pl (R.G.); 2 Perioperative Medicine, Barts Heart Centre and St. Bartholomew’s Hospital, London EC1A 7BE, UK; 3 Department of Anesthesiology and Critical Care, Policlinico Umberto I, “Sapienza” University of Rome, 00161 Rome, Italy; f.pugliese@uniroma1.it (F.P.); bilotta@tiscali.it (F.B.)

**Keywords:** postoperative pain, pain management, acute pain, liver resection, hepatectomy

## Abstract

Background: The existing recommendations for after open liver surgery, published in 2019, contains limited evidence on the use of regional analgesia techniques. The aim of this systematic review is to summarize available clinical evidence, published after September 2013, on systemic or blended postoperative analgesia for the prevention or treatment of postoperative pain after open liver surgery. Methods: The PUBMED and EMBASE registries were used for the literature search to identify suitable studies. Keywords for the literature search were selected, with the authors’ agreement, using the PICOS approach: participants, interventions, comparisons, outcomes, and study design. Results: The literature search led to the retrieval of a total of 800 studies. A total of 36 studies including 25 RCTs, 5 prospective observational, and 7 retrospective observational studies were selected as suitable for this systematic review. Conclusions: The current evidence suggests that, in these patients, optimal postoperative pain management should rely on using a “blended approach” which includes the use of systemic opioids and the infusion of NSAIDs along with regional techniques. This approach warrants the highest efficacy in terms of pain prevention, including the lower incretion of postoperative “stress hormones”, and fewer side effects. Furthermore, concerns about the potential for the increased risk of wound infection related to the use of regional techniques have been ruled out.

## 1. Introduction

Pain after open liver surgery can be attributed to two major mechanisms: peripheral nociceptors stimulation (induced by subcostal incision, rib retraction, diaphragmatic irritation, etc.) and visceral origin transmitted by sympathetic nerves [1,2,3]. Due to its multifactorial origin, it remains a major clinical challenge and current evidence suggests that adequate postoperative pain relief is accomplished in only 20–45% of patients undergoing major abdominal surgery [4,5,6,7,8]. Many perioperative strategies have been evaluated in this group of patients but there is still no consensus on the best practice [9,10]. Commonly used modalities for postoperative pain control are systemic intravenous analgesics, epidural analgesia, and peripheral nerve blocks [11,12]. Systemic intravenous administration of analgesics (as opioids and non-steroidal anti-inflammatory drugs) is effective but associated with potentially harmful side effects, such as respiratory depression, nausea and vomiting, pruritus, gastrointestinal bleeding, and renal failure [13,14]. Epidural analgesia might provide improved pain control, but in the specific setting of open liver surgery, may potentially challenge and have harmful drawbacks as a sympathetic blockade (with hypotension, bradycardia) and intraoperative fluid overload arises and its use might be limited because of the possible perioperative coagulopathy [13,15,16,17,18]. A peripheral nerve block, such as the paravertebral nerve block (PVB), transversus abdominis plane block (TAP), or quadratus lumborum block (QLB), performed under ultrasound guidance is an emerging alternative that could provide effective analgesia with a potentially low-risk profile after open liver surgery.

In 2015, a comprehensive review, that included fourteen studies published between November 1966 and September 2013, reported the superiority of epidural analgesia in pain relief over alternatives, but without translating into a reduction in length of hospital stay (LOS) and postoperative complication rates [19]. The existing recommendation from ESRA for Procedure-Specific Postoperative Pain Management (PROSPECT) after open liver surgery published in 2020 contains limited evidence on the use of regional analgesia techniques in this setting [20]. Since then, new relevant clinical evidence has emerged, and clinical practice is evolving accordingly.

The aim of this systematic review (SR) is to summarize the available clinical evidence, published after September 2013, on systemic or blended postoperative analgesia for the prevention or treatment of postoperative pain after open liver surgery.

## 2. Materials and Methods

### 2.1. Search Strategy

This SR was performed in accordance with the Preferred Reporting Items for Systematic Reviews and Meta-Analyses (PRISMA) statement recommendations, and the study was registered in the International Prospective Register Of Systematic Review (PROSPERO registration number CRD42020152836) [21,22]. Systematic research using PUBMED and EMBASE was performed to identify trials suitable for inclusion in this SR. Keywords for the literature search were selected, with the authors’ agreement, using the PICOS approach: participants, interventions, comparisons, outcomes, and study design [23]. The terms used as key words are listed in Appendix A.

### 2.2. Study Selection and Inclusion Criteria

The inclusion criteria were randomized controlled trials (RCTs) and prospective (POS) and retrospective (RO) studies published between October 2013 and December 2020 in the adult population (older than 18 years) on analgesia in patients who had undergone open liver surgery. Papers examining analgesia in other hepatic procedures were excluded (laparoscopic resections, liver transplant recipients, hepatic ablations, liver biopsy). Only full-text papers in the English language were considered for eligibility. Studies reporting evidence on pharmacological therapies (systemic and blended analgesia) in patients who had undergone open liver surgery that included qualitative or semiquantitative pain assessment were considered suitable for this SR.

### 2.3. Outcomes

The primary outcome measure of this SR is to report evidence in terms of the reduction in pain measured with patient-derived qualitative or semiquantitative scales referable to tested analgesic therapy. The secondary outcome measures are related to safety and clinical complication as recorded in selected studies: safety (the incidences of side effects of the tested analgesic modality), need of rescue analgesia, the total amount of opioid consumption, anesthesia recovery time, hemodynamic stability, vasopressor and fluid requirement, need for blood transfusion, postoperative complication rates, inflammatory and immune response, indicators of patients’ recovery, patient satisfaction, and LOS.

### 2.4. Data Extraction and Data Analysis

Two authors (PD, MZ) independently screened and assessed titles, abstracts, and full-text papers to identify eligible articles, with FB and PA acting as arbiters. Details of the study population, type of interventions, outcomes, and other information were extracted using a standardized data extraction form that included: study design, eligibility and exclusion criteria, duration of follow-up, randomization, blinding, number and characteristics of patients, type of surgery, and drug dose and method of administration. We reported as significant efficacy those treatments that are related to *p* < 0.05.

### 2.5. Risk of Bias

The risk of bias for all included trials was assessed according to the Cochrane Collaboration’s criteria for RCTs and non-randomized controlled trials (non-RCTs) (http://handbook.cochrane.org, accessed on 1 September 2020).

## 3. Results

**Figure 1:**
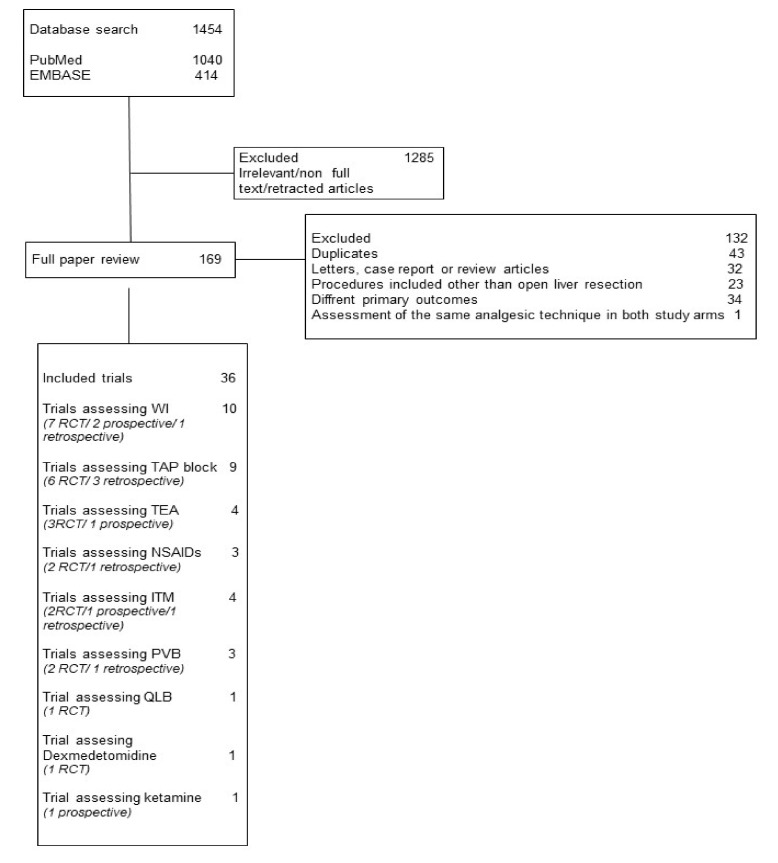
WI, wound infiltration; TAP, transversus abdominis plane; TEA, thoracic epidural analgesia; NSAIDs, nonsteroidal anti-inflammatory drugs ITM, intrathecal morphine; PVB, paravertebral block; QLB, quadratus lumborum block.

The literature search led to the retrieval of a total of 1454 studies; after the initial screening for eligibility, 1285 studies were excluded as they did not match the inclusion criteria. A total of 36 studies (involving 3560 patients, with an age range between 18 and 86) including 24 RCTs, 5 prospective observational (POS), and 7 retrospective observational (RO) studies were selected as suitable for this SR, and risk of bias was evaluated (Figure 1, Table 1 and Table 2). In 35 of the 37 studies, patients who underwent elective open liver surgery were selectively recruited, while 2 also included patients who underwent hepato-pancreato-biliary surgery; 28 studies included patients scheduled for tumor resection, and 9 studies included living donors. In the selected trials, 9 different analgesic modalities and their combinations were assessed: wound infiltration (WI), transversus abdominal plane (TAP) block, thoracic epidural analgesia (TEA), non-steroidal anti-inflammatory drugs (NSAIDs), intrathecal morphine (ITM), paravertebral block (PVB), quadratus lumborum block (QLB), dexmedetomidine, and ketamine. The evaluation of postoperative pain was assessed using various quantitative and semi-quantitative pain-rating scales and the consumption of opioid/non-opioid analgesics (Table 3). The duration of follow-up ranged from the immediate postoperative period up to 6 months after surgery. The evidence supported by the larger number of recruited patients will be displayed first.

### 3.1. Trials Assessing WI

The role of WI with local anesthetics to prevent postoperative pain after open liver surgery was evaluated in ten studies, which included a total of 1412 patients: five compared to TEA (2 RCTs, 2 POS, and 1 RO), four to placebo (RCTs), and one to systemic opioids (RCT) [24,25,26,27,28,29,30,48,49,50]. When compared to TEA, the use of WI produced conflicting evidence: two studies proved WI less effective in pain control than TEA while three studies proved no difference; of the three studies that reported data on LOS, one proved WI was associate with shorter LOS compared to TEA and two recorded no differences; no differences in surgical complication after WI or TEA were reported (Table 3) [24,25,48,49,50]. When compared to the placebo, results showed WI as being more effective in preventing pain and it reduced opioid consumption, but no differences in patient satisfaction were proven; the use of WI was associated with a less systemic release of “stress hormones” (plasma concentration of epinephrine, norepinephrine, and cortisol) than the placebo; there were no differences in the side effects rates between the two treatments (Table 3) [26,27,28,29]. In a 3-arms RCT, treatment with WI resulted in better pain control and a lower side effects or serious complications rate than assignment to systemic PCA fentanyl or tramadol. Of note, patients assigned to receive tramadol had a higher mortality rate at the 25 months follow-up than those in the two other study groups (Table 3) [30].

### 3.2. Trials Assessing TAP Block

The efficacy of a TAP block in preventing postoperative pain after open liver surgery was tested in nine studies, which included a total of 816 patients: two compared to neuraxial analgesia (2 RO), two to placebo (2 RCTs), three to systemic opioids (2 RCTs and 1 RO), one assessed the combination with NSAID-parecoxib-(RCT) and one to WI (RCT) [31,32,33,34,35,36,51,52,53]. According to results reported in an RO that included four study groups (combination of TAP block plus neuraxial analgesia; TAP block alone; neuraxial analgesia alone; or systemic opioids), patients that received a TAP block in combination with TEA showed the lowest pain scores and required less opioid consumption; those treated with the remaining approaches resulted in progressively lower efficacy (Table 3). Of note, the use of a TAP block is associated with the shortest LOS compared to the other treatments (Table 3) [51]. Data on better pain control with a TAP block vs. intrathecal morphine is also confirmed by an RO that proved lower pain scores on day 1, but no difference on the subsequent days (Table 3) [52]. When compared to the placebo, to the use of systemic opioids or parecoxib, the use of a TAP block proved to be consistently more effective in preventing pain and reducing opioid consumption (Table 3) [31,32,33,34,35,53]. Compared to WI, the TAP block was equally effective in pain control and led to lower opioid consumption (Table 3) [36].

### 3.3. Trials Assessing TEA

The effectiveness of TEA in pain management after open liver surgery was evaluated in four studies, which included a total of 393 patients: three compared to systemic opioids (2 RCTs and 1 POS) and one compared to a combination of systemic opioids and WI (RCT) [37,38,39,54]. When compared to systemic opioids, the use of TEA resulted in more effective pain control and in being associated with less opioid consumption and greater patient satisfaction with pain control (Table 3) [38,39,54]. Furthermore, the association of systemic opioids with WI provided greater pain control than TEA.

### 3.4. Trials Assessing NSAIDs

The role of NSAIDs in preventing postoperative pain after open liver surgery was evaluated in three studies, which included a total of 186 patients: two compared to placebo (RCTs) and one compared parecoxib to ketorolac (RO) [40,45,55]. When compared to the placebo, the use of NSAIDs proved to be more effective in pain control and associated with a higher opioid-sparing effect. These studies also suggest that NSAID use is possibly associated with a more preserved immune function (by increasing CD3+ and NK cell levels), reduced systemic inflammatory response (decreased levels of IL-4 and increased TGF-β), associated with a longer tumor-free interval and disease-free survival time (Table 3) [40,45]. The use of parecoxib or ketorolac leads to similar results in terms of postoperative pain control (Table 3) [55].

### 3.5. Trial Assessing ITM

The efficacy of ITM in preventing postoperative pain after open liver surgery was evaluated in four studies, which included a total of 374 patients: three compared to systemic opioids (RO and 2 RCTs) and one to TEA (PO) [42,43,56,57]. When compared to systemic opioids, the use of ITM proved to be associated with lower pain, but not the reduction in complications or the side effects of analgesia rates; no differences in functional recovery time were proven (Table 3) [42,43,56]. When compared to TEA, ITM was less effective in controlling pain during the first 12 postoperative hours, but there were no observed differences afterward, and patients receiving ITM had larger intraoperative blood loss (Table 3) [57].

### 3.6. Trials Assessing PVB

The role of PVB in preventing postoperative pain after open liver surgery was tested in three studies, which included a total of 150 patients: one compared to TEA (RCT), one to placebo (RCT), and one to systemic opioids (RO) [41,44,58]. When compared to TEA, bilateral PVB was less effective in preventing pain; the two groups had a similar rate of side effects, associated complications, rate of ICU admission, and LOS (Table 3) [44]. When compared to the placebo, the use of the right PVB was superior for pain control and was also associated with greater patient satisfaction; there were no differences reported in side effects, associated complications, and LOS between the study groups (Table 3) [41]. When compared to systemic opioids, the use of the right PVB was associated with lower opioid consumption, but there were no differences in pain scores and there were similar rates of side effects between the two study groups (Table 3) [58].

### 3.7. Trial Assessing QLB

The effects of continuous QLB in preventing postoperative pain after open liver surgery was evaluated and compared to systemic opioids in 1 RCT that enrolled a total of 63 patients [46]. When compared to systemic opioids, the use of QLB resulted in more effective pain control, shorter recovery time from anesthesia, and earlier independent mobilization after surgery; no differences in the rate of side effects were recorded between the two treatment groups (Table 3).

### 3.8. Trial Assessing Dexmedetomidine

Adding dexmedetomidine to pain management after open liver surgery was tested in one study (RCT) and compared to a placebo in a total of 52 patients [47]. Dexmedetomidine infusion, started at anesthesia induction and continued for 48 postoperative hours, was associated with better pain control than the placebo (Table 3) [47].

### 3.9. Trial Assessing Ketamine

Differences in ketamine administration routes, intravenous vs. epidural, were tested to prevent pain after open liver surgery in one study (POS) which included a total of 44 patients [59]. The two tested approaches resulted in similar efficacy in controlling postoperative pain. Of note, in patients that received intravenous ketamine, there was a higher rate of cognitive side effects: hallucinations, acute confusional syndrome, and nightmares (Table 3) [59].

## 4. Discussion

This SR is intended to update a previous review and PROSPECT guidelines on postoperative analgesia in open liver surgery and includes studies published between September 2013 and December 2020. Current evidence suggests that, in these patients, optimal postoperative pain management should rely on using a “blended approach” that includes the use of systemic opioids and NSAID infusion along with regional techniques (WI, TAP blocks, TEA, ITM, PVB, QLB). This approach warrants the highest efficacy in terms of pain prevention, including lower incretion of postoperative “stress hormones”, and fewer side effects. Furthermore, concerns on the potential for the increased risk of wound infection related to the use of regional techniques have been ruled out.

Compared to the evidence reported in the review published by Hughes and Coll [19], there are several notable differences: it is now clear that the systemic infusion of analgesics (opioids and NSAIDs) is an essential component of postoperative analgesia and there are specific indications for the most appropriate prescription considering that opioids should be better used as PCA and NSAIDs according to a pre-scheduled, TEA and other regional approaches, WI and TAP blocks, associate with similar effectiveness. Furthermore, when compared to the PROSPECT recommendation of ESRA for pain management after open liver resection, new evidence has emerged about the efficacy of QLB, dexmedetomidine infusion, and few benefits in ITM utilization in these settings [20]. As with other subspecialty procedures, postoperative pain management should be addressed considering the specific evidence-based principles [60,61,62]. This SR now provides a detailed and comprehensive summary of specific clinical evidence targeted to postoperative analgesia in open liver surgery. Of note, it is important to consider that implementing TEA into routine clinical management is affected by the so-called “team approach” [63]. It means that when an experienced team (surgeon, anesthesiologist, perioperative nurses, pharmacists, physical and respiratory therapist) takes care of patients it improves the functional outcome.

The present SR is intended to update a previous review article and has unique methodological features. First, the current SR presented was conducted in accordance with the PRISMA guidelines as with the previous one but was also approved and recorded by the PRISMA board. Data extraction was accomplished, with a dedicated data extraction form, using a PICOS approach intended to identify specific primary and secondary end points that namely included the efficacy and safety of the tested approaches. The current primary end point was selected to report on the efficacy of the tested therapies, thus, introducing a distinct perspective when compared to the previous review that primarily reported an “overall systemic complication rate”. The previous approach led the authors to find no differences among the reported approaches, while in the present SR the benefits of a blended approach are shown, as is the superiority of the pre-scheduled administration of NSAIDs, while opioids should be better prescribed as PCA. As secondary end points, the previous review reported LOS and pain scores at 24 postoperative hours both at rest and when moving proved unbeneficial. In the present SR, safety was the main focus of the selected secondary end points, and collected evidence excluded the potential for additional risk associated with regional analgesic strategies. Studies that included patients who underwent liver transplants have been excluded from the present SR because it was selectively intended to present the clinical management of patients undergoing an elective surgical procedure.

This SR has several limitations including the methodological approach that relied on the literature search being limited to two databases (PubMed and EMBASE). Despite the potential for having missed other published studies, it is appropriate to underline that the most prestigious journals are referred to in these databases and therefore the risk of omitting major information was limited. Another possible limitation refers to the exclusion of studies that presented mixed cases of open, laparoscopic, and percutaneous procedures. This might have prevented adding details collected in some studies however enabled the selection of evidence from patients with a more homogeneous clinical background. Of note, most of the studies in each treatment section have widely different design and clinical relevance. As a result of this and other shortcomings, there are often contradicting results. We acknowledge that utilizing the GRADE approach to evaluation might have added strength to data extraction. Nevertheless, as mentioned among the study’s limitations, the limited number and heterogeneity of retrieved studies prevent reaching ultimate conclusions and the present systematic review should be considered as an updated summary of available evidence and the proposal for future studies.

In conclusion, the latest evidence on postoperative analgesia in open liver surgery provides new relevant information on the effectiveness and safety of various tested approaches and supports “blended” approaches with systemic analgesic infusion (opioids and NSAIDs) along with regional techniques. Patients undergoing open liver surgery have unique clinical problems and requirements that include: the risk for hemodynamic instability due to intraoperative vascular clamping and fluid shift, potential postoperative pulmonary complications, coagulation disturbances, and metabolic abnormalities after excessive parenchymal resection, altered drugs metabolism, etc. Blended multimodal approaches were associated with the highest effectiveness and the least side effects. The NSAIDs should be better administered on a pre-scheduled structured prescription and opioids have the highest efficacy/safety profile when administered as PCA. Continuous opioid infusion, along with scheduled NSAID administration, emerged as a possible treatment after the careful analysis of available clinical evidence in this setting. None of the referenced studies specifically tested this approach that is a part of the new information presented in this systematic review as possible future research proposals. Future studies should also be addressed to identify how postoperative analgesia can effectively contribute to shortening LOS and improving functional recovery of physical and cognitive abilities.

## Figures and Tables

**Table 1:** Risk of bias for the randomized controlled trials included.

Authors	Sequence Generation	Allocation Concealment	Blinding of Participants, Personnel and Outcome Assessor	Incomplete Outcome Data	Selective Outcome Reporting	Others Criteria
Hughes et al. [24]	U	U	H	L	L	H
Bell et al. [25]	L	L	H	L	L	L
Dalmau et al. [26]	L	L	L	L	L	L
Peres-Bachelot et al. [27]	L	L	L	U	L	U
Sun et al. [28]	L	U	L	L	L	L
Xin et al. [29]	L	L	L	L	L	L
Wu et al. [30]	L	L	U	L	L	L
Karanicalas et al. [31]	L	L	L	L	L	L
Guo et al. [32]	L	L	L	L	L	L
Kıtlık et al. [33]	L	L	L	L	H	U
Erdogan et al. [34]	L	L	H	L	H	U
Qiao et al. [35]	L	L	H	L	H	L
Su et al. [36]	U	L	U	L	H	H
Hausken et al. [37]	L	U	H	L	L	L
Aloia et al. [38]	U	U	H	L	L	L
Aydogan et al. [39]	U	U	U	L	L	U
Wang et al. [40]	L	L	L	L	L	L
Chen H et al. [41]	L	L	L	L	L	U
Dichtwald et al. [42]	L	L	U	H	L	U
Niewiński et al. [43]	L	U	U	L	L	H
Schreiber et al. [44]	L	L	H	L	L	U
Chen MT et al. [45]	L	L	U	L	L	L
Zhu et al. [46]	L	U	H	L	L	L
Zhang et al. [47]	L	L	L	L	L	L

L, low risk of bias; H, high risk of bias; U, unclear risk of bias.

**Table 2:** Risk of bias for the non-randomized controlled trials included.

Authors	Bias Due to Confounding	Bias in Selection of Participants into the Study	Bias in Measurement of Interventions	Bias Due to Departures from Intended Interventions	Bias due to Missing Data	Bias in Measurement of Outcomes	Bias in Selection of the Reported Result
Wong-Lun-Hing et al. [48]	U	U	L	M	S	L	L
Khan et al. [49]	L	M	L	L	H	L	L
Che et al. [50]	S	S	L	L	L	U	L
Hernandez et al. [51]	S	S	S	U	M	L	L
Amundson et al. [52]	S	S	S	M	S	M	L
Maeda et al. [53]	S	S	S	L	U	L	L
Ganapathi et al. [54]	U	U	M	N.A	U	M	M
Lim et al. [55]	L	L	L	U	L	M	L
Tang et al. [56]	S	S	U	U	L	L	L
Kasivisvanathan et al. [57]	L	L	L	L	L	L	M
Mistry et al. [58]	S	S	S	U	S	M	M
Masgoret et al. [59]	L	U	L	N.A	M	L	L

L, low risk of bias; M, moderate risk of bias; S, serious risk of bias; C, critical risk of bias; U, unclear risk of bias; N.A, not applicable.

**Table 3:** Summary of the studies included in this systematic review.

Authors	Surgery/ Operation	Study Type/ Number of Patients (*N*)/ Tested Analgesic Techniques and Doses	Postoperative Follow-Up	Primary Endpoint	Secondary Endpoint	Key Message
Wong-Lun-Hing et al.	Elective open liver resection	Prospective study *N* = 498 WI: *n* = 429 At the end of surgery, 10 mL bolus of 0.25% bupivacaine, and then 0.25% bupivacaine, at a rate of 3 mL was continued for 72 h. Additionally IV-PCA with morphine or fentanyl. TEA: *n* = 69 During surgery 20 mL of 0.25% bupivacaine and then 0.1% bupivacaine with 2 µg/mL fentanyl at a rate of 5–15 mL/h for 48 h.	Postoperative days: 1, 2, 3	VRS score at rest and on movement during the first 48 h Total opioid consumption	Need for rescue opioid Side effects of analgesia	No differences in VRS scores between the groups. Lower total opioid consumption in the WI group than in the TEA group. Increased need for rescue opioid in WI group on PoD0 than in the TEA group. Higher sedation scores on PoD0 in WI group than in TEA group. No differences in other side effects of analgesia rate between the groups. No reported cases of epidural hematoma, abscess formation, or paralysis in the TEA group. No difference in complications rate. Shorter LOS in the WI group than in the TEA group.
Khan et al.	Living donor hepatectomy	Retrospective study *N* = 319 WI*: n* = 84 At the end of surgery a bolus of 0.125% bupivacaine, 0.2 mL/kg per side. Postoperatively, a bolus of bupivacaine 0.125%, 0.2 mL/kg per catheter twice a day. Additionally IV-PCA with morphine. TEA: *n* = 68 Intraoperatively 0.1% bupivacaine with 0.015 mg/mL hydromorphone at a rate 5 mL/h. Postoperatively 0.1% bupivacaine solution with 0.015 mg/mL hydromorphone at an infusion rate of 5 mL/h with 3 mL bolus IV-PCA: *n* = 167 with morphine or hydromorphone.	Postoperative hours: 6, 12, 18, 24, 30, 36, 42, 48, 54, 60, 66, 72	NRS scores Total opioid consumption	Side effects of analgesia: PONV, sedation, pruritus. Time to full diet Time to ambulation LOS	Higher NRS scores in the WI group than in the TEA group and similar to the IV PCA. Lower total opioid consumption in the WI group than in the IV-PCA group. Lower incidence of pruritus and sedation in the WI group compared to the TEA group. No differences in time to ambulation, incidences of PONV, and LOS between the groups.
Hughes et al.	Elective open liver resection	RCT *N* = 95 WI*: n* = 49 At the end of surgery 40 mL 0.125% levobupivacaine and then 0.375% levobupivacaine at a rate of 4 mL/h for 48 h. TEA: *n* = 44 10 mL levobupivacaine with 100 µg of fentanyl to establish epidural block than an infusion of 0.1% levobupivacaine with 2 µg/mL fentanyl for 48 h	Postoperative hours: 2, 6, 12, 24, 48, 72	Functional recovery time: independent mobilization, tolerating full diet and oral analgesics, blood tests normal and patient willing to go home.	VAS score at rest and on movement complication rates inflammatory response CVP during transection	No differences in VAS scores, morbidity, inflammatory response, and CVP during transection between the groups. Greater opioid consumption in the WI group up to PoD 1. After PoD 1 TEA group received a greater amount of opioids. Shorter functional recovery time in the WI group than in the TEA group. WI group spent less time in the HDU than the TEA group. Greater volume of iv crystalloid being administered in the TEA group on PoD 1.
Bell et al.	Elective open liver resection and living donor hepatectomy	RCT *N* = 83 WI*: n* = 42 20 mL 0.5% bupivacaine bolus followed by an infusion of 0.25% bupivacaine at 4 mL/h per catheter for 60 h. Additionally IV-PCA with morphine or oxycodone for breakthrough pain. TEA: *n* = 41 0.15% bupivacaine with 2 µg/mL of fentanyl at 6–10 mL/h and continued for 60 h postoperatively.	Postoperative hours: 6, 24,36, 48, 60	LOS	NRS score at rest Functional recovery time Peak flow Vasopressor and fluid requirements Complication rates	Higher NRS scores on PoD 0, afternoon of PoD 1, and morning of PoD 2 in the WI group than in the TEA group. Greater opioid consumption in the WI group on PoD 0, 1, and 2. No differences in side effects of analgesia and complication rates between the groups. No difference in LOS and functional recovery time between the groups. No difference in the volume of intraoperative fluid between the groups. Vasopressor support often required in the TEA group. No difference in baseline peak flow between the groups, but change in peak flow from the baseline level was worse in WI group.
Che et al.	Elective open liver resection	Prospective study *N* = 80 WI: *n* = 10 300 mL of 0.4% lidocaine for 72 h TEA: *n* = 22 Infusion of 0.2% ropivacaine at 4 mL/h with a bolus of 4 mL IV PCA: *n* = 48 morphine 0.25 mg/mL or sufentanil 0.6 mcg/mL at a rate of 4 mL/h, with a bolus of 4 mL.	Postoperative hours: 4, 12, 48, 72	VAS score at rest and on movement	Need of rescue analgesic side effects of analgesia complication rates	No difference in the VAS scores at rest or on movement between the WI group and all other groups. Higher incidences of need for rescue analgesia at 4, 12 h after surgery in the WI group than in the TEA and IV-PCA groups. No differences in the incidence of PONV and functional recovery time between the groups. No severe adverse effects associated with WI.
Dalmau et al.	Elective open liver resection	RCT *N* = 99 WI*: n* = 53 0.23% ropivacaine at 5 mL/h for 48 h. Control group: *n* = 46 with placebo In both groups: before abdominal closure, iv 50 mg of dexketoprofen, iv acetaminophen (1 g every 6 h) and iv 0.05 mg/kg of morphine. Postoperatively: IV-PCA pump with morphine, iv 50 mg of dexketoprofen and iv 1 g of acetaminophen twice a day during the 48 h.	Postoperative hours: 0, 6, 12, 24, 48	Total opioid consumption	NRS score Perioperative blood transfusion time to sit in a chair and walk Time to solid-food intake Side effects of analgesia wound complication LOS	Lower NRS score at 6 h in the WI group than in the control group. No differences in opioid consumption between the groups. No difference in transfusion requirements, solid food intake, ambulation, or LOS between the groups. Patients in the control group could sit in a chair earlier than those in the WI group. No wound complication was recorded.
Peres- Bachelot et al.	Elective open liver resection	RCT *N* = 85 WI: *n* = 42 40 mL of 0.375% ropivacaine on closure of the wound, followed by 8 mL/h continuous infusion of 0.2% ropivacaine for 96 h Control group: *n* = 43 with placebo Intraoperatively continuous iv remifentanil infusion in both groups. Acetaminophen 1 g and single morphine dose (0.2 mg/kg) were administered 1 h before the end of surgery. Postoperative analgesia: IV-PCA with morphine in both groups. Acetaminophen and nefopam as a rescue analgesia.	Postoperative hours: first 96	Total opioid consumption	VAS scores daily opioid, acetaminophen, and nefopam consumptions Time of recovery of GI function Adverse events (PONV, psychiatric disorders, tachycardia, hypotension, residual pain, wound status) LOS	No difference in VAS scores, pain management satisfaction, hemodynamic parameters, recovery of GI function, wound complications, and LOS between the groups. Lower opioid consumption in WI group compared to placebo. Lower acetaminophen requirement during the first 96 postoperative hours in the WI group compared with the control group. No difference in the consumption of nefopam during the 96 postoperative hours between the groups.
Sun et al.	Elective open liver resection	RCT *N* = 53 WI: *n* = 26 Single shot of 20 mL of 0.75% ropivacaine at the end of the surgery Control group: *n* = 27 with saline Postoperative analgesia for both groups: IV-PCA with sufentanil at a rate of 2 μg/h and a bolus of 0.5 μg.	Postoperative hours: 0, 6, 12, 24, 48	VAS score at rest and on movement	Total opioid consumption, MAP, HR, time to bowel recovery LOS PONV Concentration of epinephrine, norepinephrine, and cortisol in serum plasma	Lower VAS scores at rest and on movement at 0, 6, and 12 h postop in the WI group compared with the control group. No differences in VAS scores at rest and on movement at 24 h and 48 h between the two groups. Lower MAP and HR, total opioid consumption, shortened time to bowel recovery and LOS in the WI group than in the control group. No difference in the incidence of PONV between the groups. Lower levels of epinephrine, norepinephrine, and cortisol in the WI group than in the control group.
Xin et al.	Elective open liver resection	RCT *N* = 39 WI: *n* = 19 At the end of surgery 20 mL of 0.5% ropivacaine and then 0.3% ropivacaine at a rate of 2 mL/h per side. Control group: *n* = 20 with placebo Postoperative analgesia for both groups: PCA with sufentanil with no constant infusion	Postoperative hours: first 48	NRS score at rest and on movement	Opioid consumption PONV Sedation score Time to bowel recovery Liver function change Patient satisfaction LOS	Lower pain scores at rest after 8 and 16 postoperative hours in the WI group than in the control group. No differences in NRS scores on movement at any time in postoperative period between the groups. Reduced opioid consumption, time to bowel recovery, incidences of PONV and LOS in the WI group. Comparable sedation score and liver function change in the groups. No differences in patient satisfaction between the groups.
Wu et al.	Elective open liver resection	RCT *N* = 60 WI: *n* = 20 50 mL 0.25% ropivacaine on closure of the wound followed by 5 mL/h constant flow for 48 h IV-PCA: *n* = 20 With fentanyl Control group: *n* = 20 with tramadol injection according to the NRS scoring system.	Postoperative hours: 6, 12, 24, 48	NRS score	Side effects of analgesia Hepatic dysfunction (ALT value) Indicators of rehabilitation Wound healing	Lower NRS scores, reduced rate of analgesic usage, ambulation time, and GI function recovery time in the WI group than in the control group. Lower NRS scores at 12 postoperative hours in WI group than in IV-PCA group with no differences at the later time points. Increased mean survival time in WI and IV-PCA groups than in the control group. More side effects of analgesia and hepatic dysfunction in the IV-PCA group than in the WI and control groups with no differences between the WI and the control group. Higher incidences of incision exudation in the WI group than in the IV-PCA group and the control group.
Hernandez et al.	Elective open liver resection	Retrospective study *N* = 232 TAP block alone: *n* = 16 Open TAP block 30 mL 0.25% bupivacaine and 20 mL 1.3% liposomal bupivacaine Neuraxial (TEA or intrathecal opioid) alone: *n* = 66 Intrathecal hydromorphone 75–150 µg or TEA an infusion of bupivacaine 0.075% + hydromorphone 5 µg/mL at 8–12 mL/h. IV-PCA alone: *n* = 35 Combined neuraxial + TAP block: *n* = 115	Postoperative hours: 24, 48	Median NRS score	Opioid consumption Time to first postoperative opioid administration PONV Complications related to the neuraxial anesthetics LOS	Lower NRS scores in the patients with TAP block than in the IV-PCA alone and neuraxial alone groups. Higher NRS scores in the group with systemic opioids alone than in the other groups. Lower NRS score in TEA+ TAP block than in intrathecal+ TAP block group. Lower opioid consumption in TAP + TEA/intrathecal than in systemic opioid alone group. No difference in incidences of PONV in the first 24 h between the groups. No differences in rate of complications related to the neuraxial technique between TEA+ TAP and intrathecal+ TAP. Longer LOS in systemic opioid alone group.
Amundson et al.	Living donor hepatectomy	Retrospective study *N* = 77 TAP block: *n* = 29 liposomal bupivacaine (266 mg) mixed with 30 mL of 0.25% plane bupivacaine. Control group: *n* = 48 Both groups received 100–150 µg intrathecal hydromorphone and 800 mg oral gabapentin.	Postoperative days: 0, 1, 2, 3, 4	NRS score on PoDs 0 and 1	NRS score on PoDs 2, 3, and 4. Opioid consumption Treatment of PONV Time to ingestion of clear fluids and a full diet Time to bowel activity LOS	Lower NRS scores and lower opioid consumption on PoD 0 in the TAP block group than in the control group with no differences on subsequent days. Shorter time to full diet, first bowel movement, and flatus in the TAP block group than in the control group. No differences in LOS and incidences of PONV between the groups.
Karanicolas et al.	Elective open liver resection	RCT *N* = 153 MOTAP block: *n* = 71 40 mL of 0.3% ropivacaine and then 5 mL 0.2% ropivacaine through each catheter for 72 h Control group: *n* = 82 with placebo. In both groups: IV-PCA and celecoxib	Postoperative hours: 12, 24, 36, 48, 60, 72	Total opioid consumption over the first 48 postoperative hours	NRS score at rest and on coughing Complications rates LOS	Lower NRS at rest and with coughing at all time points in MOTAP block group than in the control group. Lower opioid consumption in MOTAP block group than in the control group. Shorter LOS in MOTAP block group than in the control group. No difference in complications rate between the groups.
Guo et al.	Elective open liver resection	RCT *N* = 70 OSTAP block: *n* = 35 40 mL of 0.375% ropivacaine Control group: *n* = 35 with placebo In both groups: Intraoperatively sufentanil and dexmedetomidine with a loading dose iv administration. Postoperatively IV-PCA with sufentanil and iv parecoxib.	5 min after extubation and postoperative hours: 2, 4, 12, 24, 48	Total opioid consumption 24 h after surgery	NRS score at rest and on coughing PONV Time to extubate Side effects of analgesia	Lower NRS scores at rest at 2 h and 4 h postoperatively, and on coughing at all time points in the OSTAP block group than in the control group. Lower opioid consumption at all time points up to 24 h in OSTAP block group. No difference in opioid consumption at 48 h between the groups. Lower incidence of PONV between 4 h and 8 h in OSTAP block group. Reduced extubation time OSTAP block group. No difference in complications rates between the groups.
Kıtlık et al.	Living donor hepatectomy	RCT *N* = 50 TAP block: *n* = 25 1.5 mg/kg of 0.5% bupivacaine with saline to reach a total volume of 40 mL. IV-PCA: *n* = 25 In both groups: Intraoperatively remifentanil infusion. IV-PCA with morphine and iv acetaminophen postoperatively.	Postoperative hours: 0, 2, 4, 6, 12 and 24	Total opioid consumption	VAS score at rest and on movement sedation scores PONV Need for antiemetic medication	Lower VAS scores at rest and movement and lower opioid consumption in the TAP block group than in the control group. No difference between the two groups in terms of PONV and sedation scores.
Erdogan et al.	Living donor hepatectomy	RCT *N* = 44 TAP block: *n* = 22 1.5 mg/kg of 0.5% bupivacaine with saline to reach a total volume of 40 mL. IV-PCA: *n* = 22 Intraoperatively remifentanil infusion. In both groups: Postoperatively IV-PCA and iv acetaminophen.	Intraoperative and postoperative 24 h	Intraoperative and postoperative opioid consumption.	Difference in mean MAP and HR Anesthesia recovery time LOS	Reduced perioperative and postoperative opioid consumption in TAP block group No difference in HR and MAP between groups at any time. Shorter anesthesia recovery time in TAP group. Shorter LOS in TAP block group than in the control group.
Maeda et al.	Living donor hepatectomy	Retrospective *N* = 32 TAP block: *n* = 16 0.25% levobupivacaine 10 mL for each side at the end of surgery and then infusion of 0.125% levobupivacaine at 6 mL/h per side for 48 h IV-PCA: *n* = 16 With fentanyl In both groups: IV-PCA	Postoperative hours: 3, 6, 12, 24, 48	Total opioid consumption	VRS score Time to rescue analgesia PONV LOS	Lower VRS scores at 3 and 6 h in the TAP block group than in the control group. No differences in VRS scores at 12, 24, 48 h between the groups. Lower opioid consumption in the TAP block group than in the control group. Longer time and lower incidence of rescue analgesia requests in the TAP block group than in the control group. Lower total number of requests for supplemental analgesia in the TAP block group than in the control group. Lower incidence of PONV in the TAP block group than in the control group. No difference in LOS between the groups.
Qiao et al.	Elective open liver resection	Single blind RCT *N* = 100 TAP block+ parecoxib: *n* = 51 40 mg of parecoxib 30 min before induction, and 150 mg of 0.375% ropivacaine with 5 mg dexamethasone, before closing the abdominal incision. Control group: *n* = 49 placebo 30 min before induction, without TAP block. In both groups: Postoperatively IV-PCA with sufentanil and 40 mg of parecoxib every 12 h for 72 h.	Postoperative hours: 24, 48, and 72	VAS score at rest and on coughing	Adverse events: PONV, pruritus urinary retention, hypotension, respiratory depression. Postoperative ambulation LOS	Lower VAS scores in the study group than in the control group. No differences between the groups in terms of adverse events. Improved ambulation in the study group than in the control group. Shorter LOS in the study group than in the control group.
Su et al.	Elective open liver resection	RCT *N* = 58 TAP block: *n* = 29 open TAP block 0.2% Ropivacaine 10 mL per side. WI: *n* = 29 Subcutaneous injection of 20 mL of 0.2% ropivacaine at the incision. In both groups: postoperatively IV-PCA with sufentanil	Postoperative hours: 24, 48	Total opioid consumption	VAS score Time to first flatus PONV LOS	No differences in VAS score between the groups. Lower opioid consumption in the first 24 postoperative hours in the TAP group than in the WI group. Shorter time to first flatus in the TAP block group than in the WI group. No differences in incidence of PONV and LOS.
Hausken et al.	Elective open liver resection	RCT *N* = 143 TEA: *n* = 77 0.1% bupivacaine with fentanyl 2 mcg/mL and epinephrine 2 mcg/mL at a rate of 5 to 15 mL/h, with 2 boluses of 5 mL allowed per hour. iv acetaminophen (1 g every 6 h) IV-PCA*: n* = 66 with Ketomebidone 1 mg boluses with an 8 min lockout interval (max. 7 mg/h) with no basal infusion. iv acetaminophen (1 g every 6 h) and iv ketorolac (30 mg every 8 h) on POD 0 to POD 2. Wound infiltration with 20 mL 0.5% bupivacaine before skin closure	Postoperative days: 0, 1, 2, 3, 4, 5	Mean NRS score	Opioid consumption on PODs 0 to 2; side effects of analgesia, intraoperative blood loss, fluid requirements; need for vasoactive medication; days until discontinuation of TEA or IV-PCA; time in operating room and PACU; surgical complications, LOS	No difference in mean NRS score between the groups. Lower NRS scores in the TEA group on PODs 0 and 1, but higher or equal on PODs 2 and 5 when compared to the IV-PCA group. Lower total opioid consumption in the first 3 days in the IV-PCA group. Earlier discontinuation of pumps in the IV-PCA group than in the TEA group. No difference in bleeding, intraoperative fluid requirements, and blood transfusions between the groups. No incidences of postoperative liver failure. Greater incidence of pruritus in the TEA group. Shorter LOS for patients in the IV PCA group than in the TEA group.
Aloia et al.	Elective open liver resection (*n* = 136)/pancreatic surgery (*n* = 4)	RCT *N* = 140 TEA: *n* = 106 3–10 mL of 2% lidocaine before surgical incision. Continuous infusion of bupivacaine 0.075%+ 0.5% hydromorphone at 5–8 mL/h. Postoperatively infusion rate: 5–8 mL/h. IV-PCA*: n* = 34 Intraoperative iv opioids and then IV-PCA with hydromorphone infusion with no basal rate, 0.2 mg every 10 min of demand dosing, and a 0.5 mg nursing bolus every 1 h as needed for additional pain control.	Postoperative hours: first 48	NRS/VAS scores severe pain event rates (pain scores >7)	Patient satisfaction Total opioid consumption till PoD5 Surgical complications Side effects of analgesia Patients satisfaction LOS	Lower pain scores and severe pain event rates in the TEA group than in the IV-PCA group. Lower opioid consumption in the TEA group than in the control group. Greater patient satisfaction with pain control in the TEA group than in the IV-PCA group. No difference in rates of side effects of analgesia, surgical complication rates, and LOS between the groups.
Ganapathi et al.	Elective open liver resection	Prospective study *N* = 70 TEA 0.1% bupivacaine with fentanyl 2 μg/mL infusions intraoperatively and then TEA–PCA with extra 3 mL bolus and continuous infusion rate of 3–12 mL/h. All patients received iv acetaminophen (1 g every 6 h)	Postoperative days: 0, 1, 2, 3	Success rate of epidural catheter placement	VDS score Postoperative chest infection LOS	TEA success rate of 91%. Pain relief was effective in 91% of patients with successful TEA placement. 7% had chest infection. No difference in LOS based on the success in epidural analgesia.
Aydogan et al.	Living donor hepatectomy	RCT *N* = 40 TEA: *n* = 20 Intraoperative iv remifentanil infusion and TEA with morphine 2 mg 15 minbefore the completion of surgery. Postoperatively TEA-PVA infusion with no basal infusion. IV-PCA*: n* = 20 Intraoperative iv remifentanil infusion and iv morphine 5 mg 15 min before the completion of surgery. Postoperatively IV-PCA with morphine infusion with no basal infusion.	Postoperative hours: 2, 4, 12, 24	VAS score at rest and on movement	Total opioid consumption	Lower VAS scores at rest and at movement in TEA group than in the IV-PCA group. Lower total opioid consumption in TEA group than in IV-PCA group.
Wang et al.	Elective open liver resection	RCT *N* = 80 Parecoxib+ IV-PCA: *n* = 40 40 mg parecoxib 30 min before induction, followed by 40 mg every 12 h for 48 h after surgery. Control group: *n* = 40 with saline In both groups: IV-PCA with fentanyl.	Postoperative hours: 2, 6, 12, 24, 48	VAS score at rest and on coughing	Opioid consumption, side effects of analgesia Immune response	Lower VAS scores at 2, 6, 12, and 24 h after surgery in parecoxib group than in the control group. No differences in VAS scores between the two groups at 48 h after surgery. Lower total opioid consumption in the parecoxib group than in the control group. No differences in the incidence of side effects of analgesia between the groups. Longer median disease-free survival time of patients in the parecoxib group than in the control group. No difference in overall survival between the groups. Higher percentages of CD3+ T cells at 24 h after surgery in the parecoxib group than that in control group. Higher percentages of NK cells in the parecoxib group than that of control group.
Chen MT et al.	Elective open liver resection	RCT *N* = 56 Parecoxib+ IV-PCA: *n* = 28 40 mg parecoxib before induction followed by 40 mg every 12 h and IV-PCA with sufentanil. Control group: *n* = 28 Saline+ IV-PCA with sufentanil in the same regimen	Postoperative hours: 6, 18, 30, 42, 54, 66	VAS score at rest and on movement	Opioid consumption, rescue analgesic, Side effects of analgesia Time to first flatus and exercise on floor.	Lower VAS scores on 30–54 postoperative hours, lower opioid consumption, rescue analgesic usage and rate of incidence of PONV in the parecoxib group than in the control group. No differences in time to first flatus and exercise on floor between the groups. Greater decrease in systemic erythrocyte sedimentation rate, IL-4 concentrations at 48 h after surgery in parecoxib group than in the control group. Higher level of TGF-beta after surgery in the parecoxib group than in the control group.
Lim et al.	Living donor hepatectomy	Retrospective study *N* = 50 Ketorolac+ IV-PCA: *n* = 29 PCA with morphine and ketorolac 1.87 mg/mL postoperatively. Bolus: 0.8 to 1.0 mL with the 4-h maximal dose 16 to 20 mL. Parecoxib+ IV-PCA: *n* = 21 Single dose of IV parecoxib 40 mg 30 min before the end of surgery and then plain PCA with morphine postoperatively.	Postoperative days: 1, 2, 3	VAS score	Opioid consumption, need for rescue analgesia Side effects of analgesia Satisfaction score	No difference in the VAS scores between the groups. No difference in total opioid consumption, satisfactory score, the incidence of side effects, and the need of rescue analgesia between the groups.
Tang et al.	Elective open liver resection	Retrospective study *N* = 216 ITM: *n* = 125 150–500 µg of morphine with bupivacaine and clonidine intrathecally before skin incision Control group: *n* = 91 Postoperatively both groups received PCA with morphine	Postoperative days: 1, 2, 3	Opioid consumption on POD 1	NRS scores at rest and on movement over the first 24 postoperative hours Opioid consumption on POD 1, 2 and 3. Side effects of analgesia LOS	Lower NRS scores at rest and on movement on POD 1 in the ITM group than in the IV-PCA group with no differences afterward. Lower opioids consumption on POD 1 in the ITM group than in the IV-PVA group with no differences afterward. No differences in time to full ward diet and mobilization, side effects of analgesia, complications, LOS, and 30-day readmission between the groups. Higher hospital costs in the ITM group.
Dichtwald et al.	Elective open liver resection/pancreatic surgery	RCT *N* = 49 ITM: *n* = 23 4 µg/kg of morphine intrathecally before skin incision and intraoperatively iv remifentanil infusion IV-PCA: *n* = 26 remifentanil infusion during surgery followed by iv bolus of morphine 0.15 mg/kg before the end of surgery Postoperatively in both groups: PCA with morphine	Postoperative hours: 12, 24, 36, 48, 60, 72	NRS score at rest and on coughing	Total opioid consumption Need for rescue analgesic drugs Side effects of analgesic technique Functional recovery time	Lower NRS scores in the ITM group than in the IV-PCA group. No difference in total opioid consumption between the groups. Need for additional rescue opioid often in IV-PCA group. No differences in complication and side effects related to the analgesia and recovery parameters between the groups.
Niewiński et al.	Elective open liver resection	RCT *N* = 36 ITM: *n* = 18 0.4 mg of morphine before skin incision and intraoperatively iv remifentanil infusion IV-PCA: *n* = 18 Intraoperatively iv remifentanil infusion and single dose iv morphine (0.15 mg/kg) 30 min before extubation. Postoperatively in both groups: PCA with morphine, iv acetaminophen (1 g every 6 h) and iv dexketoprofen (50 mg every 8 h)	Postoperative hours: 12, 24, 36, 48, 60, 72	NRS score at rest	NRS score on coughing Total opioid consumption Functional recovery time sedation grade Complication rate LOS	Lower NRS scores at rest at 12 and 24 h postoperative hour in the ITM group with no differences afterward and on coughing. No differences in total opioid consumption, sedation grade, complication rate, and functional recovery time. Shorter LOS in the ITM group than in the IV-PCA.
Kasivisvanathan et al.	Elective open liver resection	Prospective study *N* = 73 ITM: *n* = 37 5 µg/kg of morphine before skin incision in combination 2.5–3 mL of 0.5% heavy bupivacaine+ postoperative IV-PCA with fentanyl TEA: *n* = 36 7–10 mL of 0.125% bupivacaine with 2 mcg/mL fentanyl and then 5–10 mL/h continuous infusion. Postoperatively in both groups: Iv acetaminophen and tramadol.	Postoperative hours: 12, 24, 36, 48, 60, 72, 84, 96	LOS	VAS score on coughing Opioid requirements Blood loss CVP Fluid requirements Hemodynamic stability Time to fluid/solid intake Mobilization Quality of recovery	Lower VAS score on coughing in the first 12 postoperative hours in TEA group than in the ITM group with no differences afterward. Shorter LOS in ITM group than in the TEA group. Higher opioid consumption, intraoperative CVP and blood loss in ITM group than in the TEA group. Faster mobilization, lower iv fluid administration, and vasopressors requirement in ITM group than in the TEA group. No difference in quality of recovery and mortality and morbidity between the groups.
Schreiber et al.	Elective open liver resection	RCT *N* = 80 TEA: *n* = 41 Intraoperative iv remifentanil infusion then TEA with 0.2% ropivacaine infusion at 5–6 mL/h starting after completion of the liver resection portion of surgery and continued postoperatively with max infusion rate of 8 mL/h. btPVB: *n* = 39 0.5% ropivacaine 15 mL each side before the surgery. Intraoperative iv remifentanil infusion and btPVB with 0.2% ropivacaine infusion at 7 mL/h each side starting after completion of the liver resection portion of surgery and continued postoperatively with max infusion rate of 12 mL/h each. Postoperatively in both group: IV-PCA with hydromorphone to maintain VRS < score 6. Adjuvant analgesics: iv ketorolac, acetaminophen, or low-dose ketamine infusion (5–10 mg·h^−1^)	Postoperative hours: 24, 48	VRS score at rest and with postoperative incentive spirometry	Total opioid consumption inspired volumes during incentive spirometry Measures of hemodynamic stability (intraoperative and postoperative fluid and vasopressor requirement) Side effects of analgesia LOS	Lower VRS score in the TEA group than in the btPVB group. No difference in total opioid consumption between the groups. No differences in rate of side effects of analgesia and LOS. No difference in maximal tidal volumes between the groups. Greater decrease in MAP 24 h-postoperatively compared with baseline in the TEA group than in the bTPVB group. No difference in vasopressive drugs and fluids given between the groups.
Chen H et al.	Elective right- lobe hepatectomy	RCT *N* = 44 Continuous right tPVB: *n* = 22 right T7 with 10 mL bolus of 0.2% ropivacaine before emergence, then continuous infusion of 6 mL/h for 24 h. Control group: *n* = 22 with saline. In both groups: remifentanil infusion during the surgery. Postoperatively IV-PCA with sufentanil.	Postoperative hours: 24	Total opioid consumption during	NRS score at rest and on coughing at 1, 4, 8, 16, and 24 postoperative hours Need of the rescue analgesia side effects of analgesia patients Satisfaction scores LOS	Lower opioid consumption at 24 postoperative hours in the PVB group than in the control group. Lower NRS scores in the PVB group than in the control group at rest and with coughing for the first 24 h. Higher patients satisfaction score in the PVB group than in the control group. No differences in the incidence of need for rescue analgesia, PONV, bloating, excessive sedation, and the LOS between the groups.
Mistry et al.	Living donor hepatectomy	Retrospective study *N* = 26 Right PVB: *n* = 16 0.2% ropivacaine infusion at a rate of 10–14 mL/h up to 7 days. Non-PVB: *n* = 10 Postoperatively both groups received PCA with hydromorphone	Postoperative hours: 24, 48, 72	Total opioid consumption	NRS score Side effects of analgesia	No difference in NRS between the groups. Lower opioid consumption in PVB group than in the control group. No difference in rates of side effects of analgesia.
Zhu et al.	Elective open liver resection	RCT *N* = 63 QLB: *n* = 32 30 min before induction 0.4% ropivacaine at 0.6 mL/kg and then ropivacaine 0.2%, at 5 mL/h continuous infusion, 5 mL bolus dose IV-PCA: *n* = 31 with sufentanil	Postoperative hours: 0.5, 2, 6, 12, 24, 48	NRS score at rest and on coughing	Time to first out-of-bed activity Self-administered analgesic counts Rate of rescue analgesia Total dose of propofol and remifentanil during surgery. Time to recovery after anesthestia	Lower NRS scores on coughing and at all time points in the QLB group than in the IV-PCA group. No differences of postoperative self-administered analgesic counts, rate of rescue analgesic usage, and incidences of analgesic-related side effects between the groups. Lower intraoperative consumption of propofol and remifentanil in QLB group. Faster recovery from anesthesia and earlier time to first out-of-bed activity QLB group.
Zhang et al.	Elective open liver resection	RCT *N* = 52 Dexmedetomidine group (DEX): *n* = 26 Dexmedetomidine infusion at an initial loading dose of 0.5 µg/kg before intubation then 0.3 µg/kg/h till the end of surgery. After surgery, for 48 h, 60 mg oxycodone and 360 µg dexmedetomidine diluted to 120 mL and administered at a bolus dose of 2 mL, with 5 min lockout interval and a 1 h limit of 20 mL. Control group*: n* = 26 with saline. Postoperatively: 60 mg Oxycodone alone with the same regimen	Postoperative hours: 1, 4, 8, 12, 24, 48	Total opioid consumption	VAS score at rest and on movement Requirement of narcotic and vasoactive drugs, haemodynamic parameters, side effects of analgesia patient satisfaction First exhaust time	Lower VAS scores at rest at 1, 4, and 8 h postoperative and with cough at 24, and 48 h after surgery in the DEX group than in the control group. Lower opioid consumption after surgery in the DEX group than in the control group. Higher patient satisfaction with pain control, shorter time to the first exhaust, and less incidence of PONV in the DEX group than in the control group. No difference in sedation between the groups. Decreased intraoperative consumption of propofol and remifentanil during surgery in the DEX group compared with the control group.
Masgoret et al.	Elective open liver resection	Prospective observational study *N* = 44 TEA with ketamine: *n* = 23 6 mL of ketamine 0.5 mg/kg + morphine 4 mg + 1% of lidocaine before surgical incision. No infusion during surgery. Before skin closure iv bolus of morphine 0.05 mg/kg and then the TEA–PCA pump 5 mL/h of ketamine 1.5 mg/mL+ morphine 15 µg/mL + ropivacaine 0.15%a till PoD5 IV-PCA: *n* = 21 iv bolus of ketamine 0.5 mg/kg before surgical incision and iv infusion of morphine 0.025 mg/kg/h during surgery iv bolus of morphine 0.05 mg/kg before skin closure and then IV-PCA 1 mL/h of ketamine 7.5 mg/mL +morphine 1 mg/mL+ ketorolac 1.5 mg/mL till PoD5 Both groups received iv acetaminophen (1 g every 6 h)	Preoperatively and postoperative hours: 2, 24, and after 7 days, 1 month, and 6 months	Persistent postoperative pain: VAS, NPSI, PCS and QST	Side effects: PONV, hemodynamic side effects (new onset arrhythmia or 20% deviation in MAP, cognitive side effects, and need for vasoactive drugs or transfusion. LOS	No differences in VAS scores between the groups. No not-controlled pain (VAS > 3) at 1 or 6 months. No difference in persistent postoperative pain incidence between the groups. Cognitive side effects were higher in IV group. No differences between the groups in other side effects. Median hospital LOS was 10 days in both groups.

WI, wound infiltration; TEA, thoracic epidural analgesia; VRS, verbal rating scale; POD, postoperative day; LOS, length of stay; NRS, numeric rating scale; PONV, postoperative nausea and vomiting; IV-PCA, intravenous patient controlled analgesia; VAS, visual analogue scale; CVP, central venous pressure; HDU, high dependency unit; GI, gastrointestinal; MAP, mean arterial pressure; HR, heart rate; TAP, transversus abdominis plane; MOTAP, medial open transversus abdominis plane; OSTAP, oblique subcostal transversus abdominis plane; PACU, post-anesthesia care unit; VDS, verbal descriptor scale, ITM, intrathecal morphine; btPVB, bilateral thoracic paravertebral block; tPVB, thoracic paravertebral block; QLB, quadratus lumborum block.

## Data Availability

Not applicable.

## Appendix A

resection* OR excision* OR surgery* OR surgery[Mesh]

AND

liver* OR liver[Mesh] OR hepatic*OR hepatectomy*

AND

epidural OR spinal OR intrathecal OR paravertebral OR erector spinae plane OR ESP OR transversus abdominis plane block OR TAP OR regional OR local OR nerve block OR wound infiltration OR wound catheter OR local infiltration OR opiate OR opioid OR systemic OR patient controlled analgesia OR PCA OR ropivacaine OR bupivacaine OR levobupivacaine OR mepivacaine OR lidocaine OR lignocaine OR xylocaine OR tetracaine OR fentanyl OR sufentanil OR remifentanil OR alfentanil

AND

pain OR analgesia OR VAS OR visual analogue scale OR postoperative pain OR pain management
